# Synaptopathy, circuitopathy and the computational biology of Huntington’s disease

**DOI:** 10.1186/s12915-018-0539-y

**Published:** 2018-06-27

**Authors:** Anthony J. Hannan

**Affiliations:** 1 Florey Institute of Neuroscience and Mental Health, Melbourne Brain Centre, University of Melbourne, Parkville, VIC Australia; 2 Department of Anatomy and Neuroscience, University of Melbourne, Parkville, VIC Australia

## Abstract

Huntington’s disease (HD) is one of the most common tandem repeat disorders and presents as a unique trilogy of cognitive, psychiatric and motor symptoms. One of the major mysteries of HD is why it selectively affects specific neuronal populations. A new article in *BMC Biology* provides a piece in the puzzle of pathogenesis. By demonstrating the delicate relationship between cortical and striatal neurons, it provokes broader questions of how we might understand HD as a disorder of synapses, neural circuits and systems biology.

## Commentary

Expansion of tandem repeat sequences led to a diversity of tandem repeat disorders defined by their repeat sequences and the genes in which they occur. Huntington’s disease (HD), which presents with a wide range of neurological, psychiatric and other symptoms [1], is one of the most common and is caused by a tandem repeat (CAG) expansion leading to an expanded polyglutamine tract in the disease protein (huntingtin). The cognitive, psychiatric and motor symptoms often occur in mid-life, when sufferers are at their prime, although juvenile onset occurs in around 5% of cases [2]. The *huntingtin* (*HTT*) gene is expressed widely throughout the brain and body and one of the major mysteries of HD is why it selectively affects specific neuronal populations, including medium spiny neurons (MSNs) of the striatum. Another mystery involves the long temporal lag between expression of the gene mutation from early development onwards and disease onset later in life.

One clue regarding the mystery of cellular selectivity in HD pathogenesis is the fact that specific cortical projection neurons form synapses onto MSNs and that this dense corticostriatal innervation could make the MSNs vulnerable to excitotoxicity, as these excitatory synapses signal via postsynaptic calcium and other signalling pathways that require tight homeostatic regulation. Whilst some of this synaptic dysregulation (synaptopathy) has been deduced via in vivo experiments [3], the use of in vitro approaches allows specific questions to be addressed.

In the present article [4], a corticostriatal primary neuron culture system was used to probe the delicate balance between cortical and striatal neurons, providing new insight into HD pathogenesis. The cortical and striatal (MSN) neurons were co-cultured from the YAC128 transgenic mouse model of HD and wild-type (WT) littermate mice. Somewhat surprisingly, reducing cortical input was seen to accentuate pathology. This could be due to reduced presynaptic release of trophic factors, including brain-derived neurotrophic factor (BDNF), at corticostriatal synapses, although this was not addressed in the present article. A key experiment demonstrated that reducing the ratio of cortical to striatal neurons in culture from 1:1 to 1:3 led to a significant loss of dendritic spines on the MSNs from the HD mice; however, this also led to a less marked impairment of dendritic length and complexity [4]. These investigators also used chimeric (HD and WT) co-cultures to demonstrate that this instability of dendritic spines was cell-autonomous and apparently driven by expression of the mutant HD transgene within the MSNs. Furthermore, this reduction of cortical input onto the MSNs from HD mice reduced their survival in vitro [4].

These findings not only help optimise this co-culture system, to facilitate future screening for drugs and other therapeutics, but also provide novel insights into the pathogenesis of HD. They also provoke broader questions as to how we might begin to dissect synaptopathy in HD, its pre- and post-synaptic components and its relationship to cell dysfunction and death. Whilst a wide variety of molecular and cellular mechanisms have been implicated in HD [2], synaptopathy appears to be one of the key pathogenic processes and provides various pre- and post-synaptic therapeutic targets [3, 5–10].

**Fig. 1.**
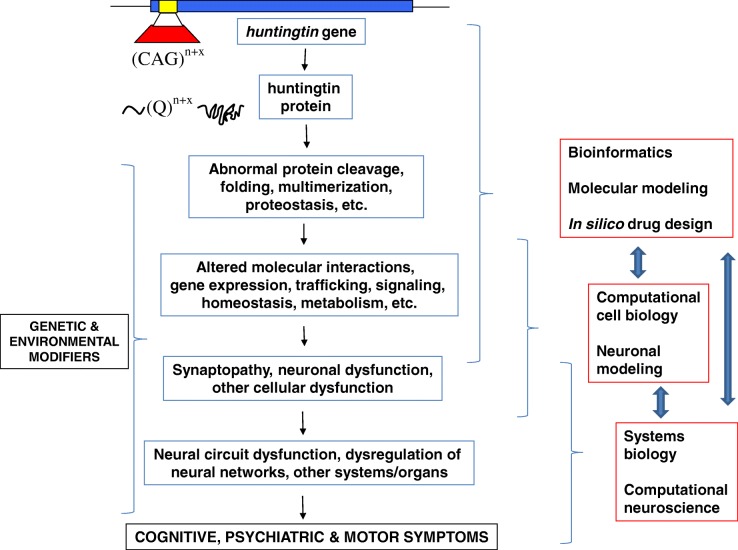
Pathogenic pathways in Huntington’s disease (HD) and the different levels at which modelling, computational approaches and systems biology might be applied to comprehensively understand pathogenesis and systematically identify therapeutic targets. HD is caused by a tandem repeat expansion mutation so that the trinucleotide (CAG) is expanded to become (CAG)^n+x^ in the *huntingtin* gene. This tandem repeat tract is transcribed and translated to become a (Q)^n+x^ polyglutamine tract in the huntingtin protein. This mutant protein leads to a cascade of molecular, cellular and systems changes which, together with the modulatory actions of genetic and environmental modifiers, ultimately lead to the onset of disease symptoms. By using more sophisticated approaches incorporating modelling, computational biology and systems neuroscience, we can obtain a more comprehensive and integrated understanding of the pathogenesis of HD, which will have major implications for therapeutic approaches aimed at preventing, treating and ultimately curing this devastating disease

In order to attempt to parse the relative components of synaptopathy, we need to consider their pre- and post-synaptic components, as well as the mediating roles of glia at tripartite synapses [3]. Key areas of focus in ongoing research should address molecular mediators of synaptic function, and the way in which affected synapses contribute to neural circuits mediating the cognitive, affective and motor functions disrupted in HD (Fig. 1). In order for a candidate molecule to be identified for therapeutic development, it should be first established that it contributes to a pathogenic pathway and is necessary for pathogenesis (the criterion of sufficiency is unlikely to be fulfilled by any single molecule). The second key question lies at a neural circuit and systems level and involves understanding which cells and synapses mediate disease endophenotypes and systems. Technologies are being developed that may increase our capacity to therapeutically target specific neural circuits and brain regions. Therefore, our understanding of HD pathogenesis must incorporate inter-cellular and intra-cellular mechanisms of pathogenesis. For example, the present study [4] not only suggests that MSNs in the striatum are exquisitely sensitive to the number of corticostrial inputs that receive from cortical projection neurons, but their pathogenic response (leading to the death of MSNs) can be cell autonomous.

Ultimately, what is required is a comprehensive computational model of the pathogenesis of HD which, as a supposedly ‘simple’ monogenic disease, could form the template for tackling more complex polygenic disorders. Whilst such an approach can be simplified to the bridging of molecular, cellular, systems and symptomatic levels (Fig. 1), the reality is far more complex and challenging. A variety of transcriptomic and proteomic approaches have probed the molecular pathogenesis of HD. Whilst a recent attempt has been made to integrate the molecular mediators of synaptopathy in HD [3], this is certainly not systematic, and much more could be done with bioinformatics and computational biology approaches to systematise this approach. Similarly, large numbers of cell populations (both central and peripheral) have been implicated in the pathogenesis of HD. Another necessary layer in this computational model would be to integrate neuronal (and glial) cell populations into neural circuits and cellular systems which are most vulnerable at early and late stages of pathogenesis.

This proposed computational model of HD pathogenesis would have far more than theoretical value. If such a model can be accurately constructed, it would lead to a range of testable hypotheses, to be explored both in vitro and in vivo, using valid preclinical models. Importantly, an accurate computational model, together with the preclinical models, could identify therapeutic targets. HD has often served as a pioneering exemplar in the field of neurodegenerative disease. This continues to be the case, as Enroll-HD has been established as one of the most powerful international clinical registries to explore the pathogenesis of HD, identify therapeutic targets and establish clinical trials. Enroll-HD (www.enroll-hd.org) is recruiting over 20,000 international participants from HD families and provides an exceptionally rich resource for international researchers. I propose that computational models of HD should be based not only on preclinical models, but also on this clinical data from presymptomatic and symptomatic subjects, which include a vast array of central and peripheral biomarkers.

More sophisticated understanding of synaptopathy and neural circuit dysfunction, as well as improved screening platforms to develop novel therapeutic approaches, are urgently needed for this devastating brain disease. HD offers unique opportunities to start at a single genetic trigger (the tandem repeat expansion) and computationally construct a pathogenic pathway from molecules to cells, circuits, systems and symptoms. This would not only facilitate new approaches to prevent, treat and eventually cure HD, but would also provide a novel template for the computational biology of disease. These kinds of preclinical and clinical strategies are required to roll out a new era of preventative, personalised and precision medicine in the twenty-first century.
